# Intrauterine Viral Infections: Impact of Inflammation on Fetal Neurodevelopment

**DOI:** 10.3389/fnins.2021.771557

**Published:** 2021-11-10

**Authors:** Sourav Ganguli, Pavithra L. Chavali

**Affiliations:** ^1^CSIR-Center for Cellular and Molecular Biology, Hyderabad, India; ^2^Academy of Scientific and Innovative Research (AcCSIR), Ghaziabad, India

**Keywords:** autism spectrum disorder (ASD), blood brain Barrier (BBB), blood CSF barrier (BCSFB), microcephaly, neurodevelopment, inflammation, cytokines

## Abstract

Intrauterine viral infections during pregnancy by pathogens such as Zika virus, Cytomegalovirus, Rubella and Herpes Simplex virus can lead to prenatal as well as postnatal neurodevelopmental disorders. Although maternal viral infections are common during pregnancy, viruses rarely penetrate the trophoblast. When they do cross, viruses can cause adverse congenital health conditions for the fetus. In this context, maternal inflammatory responses to these neurotropic pathogens play a significant role in negatively affecting neurodevelopment. For instance, intrauterine inflammation poses an increased risk of neurodevelopmental disorders such as microcephaly, schizophrenia, autism spectrum disorder, cerebral palsy and epilepsy. Severe inflammatory responses have been linked to stillbirths, preterm births, abortions and microcephaly. In this review, we discuss the mechanistic basis of how immune system shapes the landscape of the brain and how different neurotropic viral pathogens evoke inflammatory responses. Finally, we list the consequences of neuroinflammation on fetal brain development and discuss directions for future research and intervention strategies.

## Introduction

Neurodevelopment is a complex developmental process that begins during the third week of gestation and continues postnatally until adulthood (Stiles and Jernigan, 2010). In the fetal brain, about 86 billion neurons must be generated in a spatiotemporally coordinated manner, with any deviations resulting in developmental defects and cognitive impairments (Azevedo et al., 2009; Herculano-Houzel, 2009; Kang et al., 2011). The formation of neuroectoderm, which gives rise to the neural tube, is the first step in brain development. Following that, forebrain, midbrain, and hindbrain are generated in a controlled and systematic manner, developing into a functional brain (Chan et al., 2017; Elshazzly and Caban, 2019). Symmetric division of neuroepithelial cells (stem cells) that line the ventricles of the neuroectoderm results in the generation of additional neural stem cells, whereas asymmetrical division gives rise to radial glia and later differentiated cells such as neurons and various glial cells (Götz and Huttner, 2005; Subramanian et al., 2017). Newly formed neurons migrate to the cortical plate and populate distinct layers, culminating in the six-layered neocortex, which accounts for the vast majority of brain volume (Nadarajah and Parnavelas, 2002; Ayala et al., 2007). Therefore, the balance between symmetric and asymmetric cell divisions is critical in determining the brain volume and cognitive capacity (Lu et al., 2000; Huttner and Kosodo, 2005; Knoblich, 2008; Lazutkin et al., 2019). After neurons complete their migration, their projections- axons and dendrites- form synapses with appropriate synaptic partners. The earliest synaptic connections thus formed in the preplate, as early as week 5, aid in the establishment of neuronal connections from thalamus and brainstem. These early synapses contribute to the formation of more stable connections later in development (Tau and Peterson, 2010). Genetic defects, environmental perturbations or pathogens can promote mitotic defects in progenitor cells or apoptosis resulting in hypocellularity leading to disruption of the stem cell balance, which often manifests as neurodevelopmental disorders such as microcephaly and lissencephaly (Chavali et al., 2014; Heffernan and Hare, 2018). In contrast to these catastrophic failures of cell death and fate specification, changes in the neural circuit formation and functions can result in other neurodevelopmental disorders such as autism spectrum disorders (ASD) and schizophrenia.

Proliferation and programmed cell death are both vital in sculpting the landscape of a developing brain. Programmed cell death is required for (i) neural tube closure and neuroepithelial modeling (Yamaguchi et al., 2011) and (ii) to reduce replication errors by terminating cells with unfavorable genomic changes (Rehen et al., 2001; Peterson et al., 2012; Bushman and Chun, 2013). Furthermore, programmed cell death also regulates neuronal identity and patterning by eradicating population of neurons with improper projections, which might affect axonal targeting (Clarke et al., 1998; Yamaguchi and Miura, 2015). Failure to do so and eliminate surplus neurons can result in detrimental neurodevelopmental consequences (Kanold, 2009). For instance, individuals with ASD have an enlarged brain phenotype (macrocephaly) in their early years, characterized by an aberrant expansion of neocortical excitatory neurons, skewing the balance between excitatory and inhibitory circuits (Courchesne et al., 2011; Fang et al., 2014; Wei et al., 2014). On the other hand, uncontrolled apoptosis of progenitors can cause microcephaly or schizophrenia, which is characterized by localized non-lethal apoptosis, that can cause neurite and synaptic loss (Oppenheim, 1991; Chambers et al., 2004; Jarskog et al., 2005).

In this regard, the role of immune system in brain development is paramount (Zengeler and Lukens, 2021). In the sections that follow, we provide insights into how the proper functioning of the immune system is critical for brain development, and how prolonged inflammation caused by the maternal immune system in response to viral infections might jeopardize fetal neurodevelopment.

## Immune System of the Developing Brain

**FIGURE 1:**
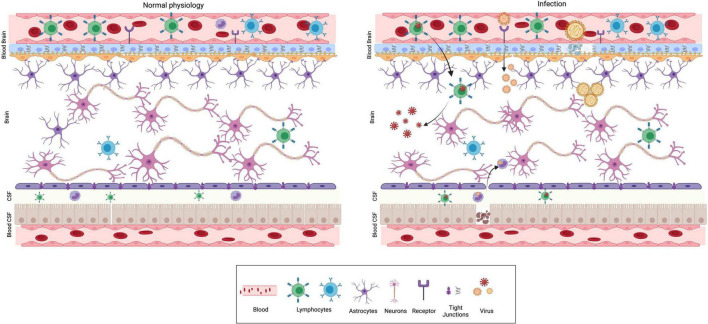
Physical barriers of the brain in normal physiology and infection. The major physical barriers to gain entry into the brain are: Blood Brain Barrier (BBB) and Blood CSF (BCSF) Barrier. BBB is composed of endothelial cells, pericytes and glial cells. The endothelial cells are held together by tight junctions, which prevent permeability and acts as a barrier. The BCSFB is composed of epithelial cells of the choroid plexus and ependymal cells. Under normal physiological conditions (left panel), pathogens, toxins, several immune modulatory molecules are barred from entering the brain by the presence of these barriers. Neurotropic viruses can breach the BBB using different mechanisms as depicted from left to right. (i) Trojan horse mechanism—virus infects peripheral immune cells, followed by the infected cell crossing BBB ultimately resulting in CNS infection. (ii) Receptor mediated transcytosis—several viruses can directly bind to receptors and cross the BBB by transcytosis. (iii) Some viruses are capable of dissolving tight junctions and compromise BBB’s protective function.

The central nervous system (CNS) and the immune system are complex organ systems which are intricately linked. Indeed, there is an evolutionary correlation in the emergence of acquired immunity and the highly developed myelin sheath in neurons (Stassart et al., 2018). The immune system of the brain comprises physical barriers such as blood-brain barrier (BBB), blood-CSF barrier (perivascular BCSFB) and an innate immune system composed of specialized non-neuronal cells known as microglia (Fenstermacher, 1980; Ballabh et al., 2004; Daneman and Prat, 2015) (Figure 1). Furthermore, in response to environmental insults, immune cells from the periphery can infiltrate *via* the lymphatic drainage portal (glial-lymphatic pathway) (Iliff et al., 2012). The presence of various physical barriers such as BBB and BCSFB indicates an evolutionarily conserved strategy for brain protection. The BBB forms as early as gestational week 8, beginning with telencephalon vascularization and progressing through coordinated cell-cell communications between CNS and the neurovascular unit during the subsequent stages (Saunders et al., 2012). The adult BBB is made up of endothelial cells, pericytes, astrocytes, microglia, and neurons. The embryonic BBB, on the other hand, lacks neurons and astrocytes but is nonetheless functional (Pardridge, 2005; Abbott et al., 2010; Daneman and Prat, 2015). The influx transporters of the BBB transport glucose, minerals, vitamins, hormones and other essential substances, through diffusion, receptor mediated transcytosis or solute carrier transporters to meet the nutrient needs of the developing brain (Roberts et al., 2008). Thus, infections or other neurotoxins breaking the BBB during pregnancy would have a detrimental impact on fetal neurodevelopment. The principal purpose of BCSFB, which is made up of cuboidal epithelium from the choroid plexus, is to secrete cerebrospinal fluid (CSF) into the brain ventricles, where it bathes neurogenic niches in nutrients (Saunders et al., 2012; Liddelow, 2015).

**FIGURE 2:**
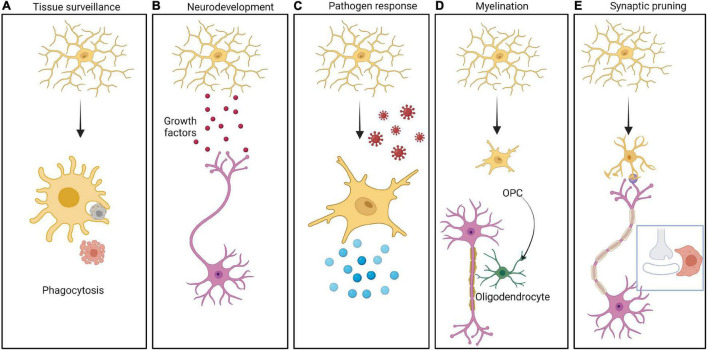
Different roles of Microglia in brain function. The CNS resident innate immune cells, namely microglia can be resting or activated in response to different stimuli. Multiple roles of microglia are depicted in the panels from left to right as follows: **(A)** Cells undergoing apoptosis in the CNS are cleared by microglia by phagocytosis. **(B)** Microglia secrete a large variety of growth factors including brain derived neurotrophic factor (BDNF), insulin like growth factor 1 (IGF-1), arginase-1 (Arg-1), nerve growth factor (NGF) etc. These molecules help in neurodevelopment as well as in CNS homeostasis. **(C)** Microglia serves as a defense against pathogens, by getting activated followed by secretion of pro-inflammatory cytokines generating an inflammatory response. **(D)** Microglia play an important role in myelinating and demyelinating neurons mediated by oligodendrocytes. **(E)** Microglia takes active part in synaptic pruning by engulfment of synapses in a complement/chemokine mediated manner.

In addition to these barriers, the innate immune cells of brain play a significant role in neurodevelopment. The various roles of the brain’s major innate immune cells, namely microglia, in neurodevelopment are well established. Microglia are brain tissue resident macrophages that arise from hematopoietic progenitors and populate the developing brain before BBB formation (Nayak et al., 2014; Colonna and Butovsky, 2017). Microglia provide tropic support to the other major cells of brain such as astrocytes and neurons and facilitate response to signals received from the surrounding neural environment (Nayak et al., 2014; Prinz et al., 2019) (Figure 2). For example, microglia maintain the homeostasis of a healthy brain by synaptic pruning, by constant monitoring of synaptic function which allows for maturation or elimination based on special complement and/or chemokine receptors found exclusively in microglia (Harrison et al., 1998; Paolicelli et al., 2011). Upon activation of the complement cascade, the effector complement C3 tags the appropriate synapse. Microglia express specific C3 receptors (C3R) which bind to the C3 fragment and phagocytose the synapse, resulting in pruning (Figure 2) (Stevens et al., 2007; Magdalon et al., 2020). Furthermore, microglia promotes (i) angiogenesis and vascularization by clearing excess vessels (Dudiki et al., 2020), (ii) proliferation and migration of neurons and glia (Aarum et al., 2003), (iii) programmed cell death of neural stem cells and neurons (Sierra et al., 2010), (iv) myelination (Traiffort et al., 2020), (v) establishment of neuronal circuits (Miyamoto et al., 2016), and (vi) the abundance of neural stem cells (Cunningham et al., 2013).

Primary adaptive immune cells (T and B cells) are less common in the brain parenchyma than microglia and are mostly found in the choroid plexus and meninges. T cells, despite their small number, play an important role in spatial learning, memory, and stress response (Kipnis, 2016; Korn and Kallies, 2017). Interestingly, Morris-water-maze test on mice showed that a distinct population of antigen positive CD4+ T cells specifically at the meninges promote learning and memory (Radjavi et al., 2014). While B cells might not be required for these functions, they do accumulate in the neonatal brain and decline with age (Tanabe and Yamashita, 2018). B cells (B1a) promote the proliferation of Oligodendrocyte precursor cell *via* IgM-Fcα-μR signaling and contribute to oligodendrogenesis (Tanabe and Yamashita, 2018). Thus immune cells not only defend against invading microbes and fight infection, but they also contribute to the brain development through the secretion of unique molecules known as cytokines.

## Cytokines in Normal Neurodevelopment

What is the source of cytokines in the fetal brain? Basal cytokine production begins in the fetal brain as early as the fifth week of gestation and is critical for normal neurodevelopment (Mousa et al., 1999; Deverman and Patterson, 2009). These cytokines are primarily secreted by microglia and astrocytes, while the role of neurons in inflammatory response is becoming more evident (Freidin et al., 1992; Hanisch, 2002; Galic et al., 2012; Stolp, 2013). Additionally, maternal cytokines can enter the fetal brain from periphery and/or *via* the placenta. Maternal cytokines can enter the brain by (i) passive diffusion bypassing the BBB where they bind to endothelial cells and induce secondary messenger signaling, (ii) endocytosis or (iii) by direct secretion at the nerve terminals (Watkins et al., 1995). Based on their role during immune response, the secreted cytokines can be broadly classified as pro- or anti-inflammatory cytokines. The major anti-inflammatory cytokines viz., IL4, IL10, IL1RA, IL11 and IL13, belong to the Interleukin (IL) family. Among the most important pro-inflammatory cytokines are the tumor necrosis factor α (TNFα), interferon γ (IFNγ), IL1 and IL18. However, cytokines can be pro- or anti-inflammatory depending on the context, as exemplified by IL6 and transforming growth factor beta (TGFβ) (Sanjabi et al., 2009; Tanaka et al., 2014). TGFβ is an important mediator of oligodendrocyte differentiation and polarizes microglia to sites of injury near pericytes (O’Keefe et al., 1999). The role of pro-inflammatory cytokines in normal neurodevelopment has come to the forefront particularly with the revelation that enhancement in the levels TNFα and IL1β coincide with major neurodevelopmental events and their roles in neuronal connectivity (Garay and McAllister, 2010; Ratnayake et al., 2013). Additionally, IFNγ in combination with TNFβ and IL1 induces the expression of the major histocompatibility complex 1 (MHC1), which is a glycoprotein expressed by all nucleated cells in the body (Raval et al., 1998). MHCs are broadly classified into two classes: Class I (ubiquitously expressed) and Class II (Antigen presenting cells). Activated MHC1 negatively regulates axon outgrowth, dendritic branching and synaptic density particularly in the hippocampal region of the brain, which is required for learning and memory (Cebrián et al., 2014). As seen with schizophrenia, any condition that triggers maternal MHC1 signaling has the potential to downregulate neuronal synapses and connectivity (Gaser et al., 2004).

Accumulating evidence suggests that a special class of cytokines called chemokines, initially thought only to attract immune cells to the site of inflammation, play a variety of roles in CNS development and in adult brain functioning (Miller et al., 2008; Trettel et al., 2020). For example, chemokine CXCR4 which evolved prior to the emergence of immune system has an important role in neural stem cell migration during injuries. CXCL12-CXCR4 influence the migration of neuronal precursors, axon guidance/pathfinding and maintenance of neural progenitor cells (NPCs) (Dziembowska et al., 2005; Li and Ransohoff, 2008). Chemokine signaling *via* CX3CL1 and CX3CR1 is neuroprotective and mediates communication between neurons and microglia, thus affecting synaptic pruning (Biber et al., 2007; Pawelec et al., 2020).

The balance between different cytokines in immune system also helps to preserve the barrier integrity. Interferons (Type1), such as IFNβ and IFNα that signal through IFNαR, are produced in response to pathogen-associated molecular patterns (PAMPs)(Zanin et al., 2021). These IFNs promote tight junction formation and preserve its integrity *via* the cytoskeletal GTPase Rac1, that promotes endothelial barrier function (Al-Sadi et al., 2009; Meunier and Broz, 2016). In contrast, IFNγ–CXCL10 signaling enhances BBB permeability due to internalization and downregulation of tight junction proteins (Daniels and Klein, 2015). Therefore, external stimuli such as viral infections that elicit different maternal cytokine secretion could have a long-standing consequence on the fetal neurodevelopment.

## Maternal Infections and Neurodevelopment

How do different viruses trigger immune response and reprogram neurodevelopment? The link between maternal infection status and fetal brain development garnered attention in late 1960s and early 1970s, when an increased infant mortality and/or high incidences of neurosensory deficiencies in newborns during the rubella epidemic was reported (Stern et al., 1969; Chess, 1971; Hutton, 2016). Children born during the rubella epidemic had delayed development and cognitive impairment. Similarly, maternal influenza infection has been linked to an increased risk of schizophrenia in adult life (Mednick et al., 1988; Kendell and Kemp, 1989). Subsequently, a number of clinical epidemiological studies revealed that a plethora of viral infections in pregnant mothers can result in neurological abnormalities ranging from encephalitis in the developing fetus to neuroprogressive and neurodegenerative diseases postnatally (Scola and Duong, 2017; Table 1).

**TABLE 1:** Inflammatory signatures of viruses and their neurological outcomes.

Virus	Host Cell entry receptor	Major CNS cells affected	Cytokine signature post infection	Neurological consequences
Cytomegalovirus (CMV)	Platelet-Derived growth factor, Neuropilin2, Olfactory receptor 14I1, Epidermal growth factor receptor (EGFR), Tetherin	Neurons, Glia, Ependymal cells, Choroid plexus	Monocyte chemoattractant protein 1 (MCP1) IL8, TNFα, IL6, CXCL11/ITAC, and CCL5/Regulated on Activation, Normal T Expressed and Secreted (RANTES), IL1B, IL10	Neurosensory loss, Focal encephalitis, Microcephaly, Seizures, Paralysis, Mental retardation, Autism spectrum disorder
Herpes Simplex Virus 1 (HSV-1)	Myelin-associated glycoprotein (MAG), Sialic- acid-binding Ig-like lectin, Non-muscle myosin heavy chain (NMHC)-IIA, Nectin 1	Hippocampal neurons, Brain stem neurons	Macrophage inflammatory protein1a (MIP1a), IL1β, TNFα, IL6, IL8, CCL5, CXCL10	Necrotizing encephalitis, Multiple sclerosis, Alzheimer’s disease
Epstein-Barr virus (EBV)	CD21, CD35	Astrocytes and Microglia	IL2, IFNγ, TNF, LTα, LTβ, CXCL10-CXCR3, CCL5-CCR5	Encephalitis, Meningitis, Cerebellitis, Polyradiculomyelitis, Transverse myelitis, Cranial and Peripheral neuropathies, Schizophrenia, Psychiatric abnormalities
Varicella-zoster virus	Mannose-6-phosphate receptor, Myelin associated glycoprotein (MAG)	Neurons	IL1, IL6, IL8, and Tumor necrosis factor alpha (TNFα)	Aseptic meningitis, Encephalitis, Cerebral infarction associated with granulomatous vasculitis, Myelitis, and Cranial polyneuropathy.
Rubella virus	Myelin oligodendrocyte glycoprotein (MOG), Signaling lymphocytic activation molecule (SLAMF1), CD46	Astrocytes, Neural progenitor cells	IL1β, IL6, TNFα	Congenital Rubella syndrome, Microcephaly, Encephalitis, Panencephalitis, Autism spectrum disorder
Mumps virus	Trisaccharide containing -2,3-linked sialic acid	Ependymal cells that line the ventricles, pyramidal cells in the cerebral cortex and hippocampus	TNFα, IL6, MCP1, CXCL10	Encephalitis, Aseptic meningitis
Influenza virus	Sialic acids (SAs) of cell surface glycoproteins and glycolipids	Hippocampal neurons in the CA1 and dentate gyrus	IL8, RANTES, MCP1, MCP3, MIP1α, IFNG induced protein 10 (IP-10) IL8, IL1B, IL6, IL18, TNFα and IFN α/ß	Neural tube defects, Hydrocephaly, Schizophrenia, Autism spectrum disorder
Human Immunodeficiency Virus 1 (HIV1)	CD4, CCR5, CXCR4	Microglia	TNFα, IFNα, IL6, IL8, IL1β, CCL2 and CCL5	Microcephaly, Slow neurodevelopment, Dementia, Increased risk of schizophrenia
Polio virus	Human poliovirus receptor (PVR) or CD155	Motor neuron cells in CNS	IL6, IL8, CXCL10, IFNß	Paralytic poliomyelitis
Severe acute respiratory syndrome coronavirus 2 (SARS-CoV-2)	Angiotensin-converting enzyme 2 (hACE2), Transmembrane protease serine 2 (TMPRSS2)	Choroid plexus cells, neurons	IL17, IL1, IL6, TNFα, IL15, IFNγ	Neurodevelopmental disorders?
Japanese Ecephalitis Virus (JEV)	Plasmalemma Vesicle Associated Protein (PLVAP), Gastrokine-3 precursor (Gkn3), C-Type Lectin Domain Containing 5A (CLEC5A), Heparansulfate, Glucose regulatory protein 78 (GRP78), Scavenger receptor I	Pyramidal neurons of the cerebrum, Purkinje cells of the cerebellum	IFNα, IL8, RANTES, IL6	Encephalitis, Paralysis, Seizures, Inability to speak, Memory loss, Impaired cognition, and other Mental disorders
Zika virus (ZKV)	TAM (AXL), Dendritic Cell-Specific Intercellular adhesion molecule-3-Grabbing Non-integrin (DC SIGN), Neural cell adhesion molecule 1 (NCAM1), Tyro 3	Neural Stem Cell (NSC), Neural Progenitor Cell (NPC)	Il1Rα, IL2, IL9, IL15, IFNγ, CXCL10, CXCL9	Microcephaly, Autism spectrum disorder
West Nile Virus (WNV)	TLR3, C type lectins, T cell Ig- and mucin domain–containing, molecule (TIM), and Tyro3, Axl, and Mertk (TAM), Natural Killer p44 (NKP44)	Neurons, Bovine microvascular endothelial (BMVE), Astrocytes, Microglia, Endothelial cell (EC)	IL1β, IL2, TNFα	Encephalitis, Depression, Memory loss and Motor dysfunction
Dengue Virus (DENV)	C-type lectin domain containing 5A (CLEC5A), TIM and TAM, Heparan sulfate, GRP78, Scavenger receptor I, Integrin αvβ3, Claudin 1, Nkp44, Laminin	Neurons, Astrocytes	IL8, IL13, MCP3, Granulocyte-macrophage colony-stimulating factor(GM-CSF) IL10, MIP1B, IFNγ, TNFα, RANTES, IL6, IL10	Encephalopathy, Acute disseminated encephalomyelitis, Myelitis, Neuritis brachialis, Stroke, Neuro thalamic complications acute hypokalemic paralysis

Herpes Simplex Virus (HSV-1) is one of the most common DNA viruses that affects almost a quarter of pregnant women worldwide during different stages of pregnancy. HSV-1 has been recognized as one of the significant causes of neurodevelopmental disabilities in children who are exposed prenatally (Straface et al., 2012). Primary infection of HSV-1 is usually asymptomatic and the risk of neonatal infection rises as the pregnancy progresses. On the other hand, HSV-2 infection is relatively uncommon but has been implicated in ASD (Straface et al., 2012). The other large DNA virus also belonging to the Herpesviridae, namely Cytomegalovirus (CMV) is a major public health issue leading to lifelong latent infection (Cheeran et al., 2009; Jackson et al., 2011). The vertical transmission risk of CMV is 30–40% of which infection during the first trimester of pregnancy is a prognostic for sensorineural loss and neuropsychological disorders such as schizophrenia in infants (Adler et al., 2007). The Human Immunodeficiency Virus (HIV) is also known to cause neurodevelopmental and neuropsychiatric disorders (Ghafouri et al., 2006; Van Rie et al., 2008).

RNA viruses, primarily of the Flaviviridae family are known to be neurotropic pathogens (Pierson and Diamond, 2020). The most recent outbreak was caused by Zika virus (ZKV), which was mildly febrile in adults but induced severe developmental defects like microcephaly in progeny (Krauer et al., 2017). Other related flaviviruses such as West Nile Virus (WNV), Japanese Encephalitis Virus (JEV) and Dengue virus (DENV) have much lower rates of vertical transmission, but are neurotropic (Li et al., 2017). Once they cross the placental barrier, viruses can breach fetal brain barriers through diverse mechanisms (Ayala-Nunez and Gaudin, 2020) (Figure 1). Polio and measles viruses (Oglesbee and Niewiesk, 2011; Ohka et al., 2012) can directly infect and lyse the endothelial and epithelial cells of the BBB and enter the brain parenchyma directly. The most common mechanism appears to be the downregulation of different tight junction proteins such as occludin and claudin that constitute the barrier. Neurotropic viruses such as WNV, HSV1 and ZKV, affect the barrier integrity by the downregulation of tight junction proteins, mediated by secretory cytokines such as TNFα (Verma et al., 2009; Chiu et al., 2020; He et al., 2020). JEV and DENV can damage the BBB by downregulating tight junction proteins by disrupting the endothelial glycocalyx (Puerta-Guardo et al., 2016). Cell free viruses such as HIV and ZKV enter the BBB *via* transcytotic or paracellular pathways, without compromising the membrane integrity (Agrawal et al., 2013; Calderón-Peláez et al., 2019). Another prevalent mode involves trojan horse like mechanism, where the virus infects lymphocytes or monocytes in the peripheral parts of the body that can migrate into the brain by paracellular or transcellular pathway to cross the BBB (Kim et al., 2003). For instance, HIV1 infected CD4+T cells, HTLV1 infected CD8+T cells and ZKV infected monocytes can all infiltrate the brain parenchyma. In the case of HSV1, the virions can also directly enter CNS *via* anterograde transport to reach the axonal shaft and tip in the neuronal periphery, where they are released (Kim et al., 2003; Ayala-Nunez et al., 2019). The more recent outbreak of SARS CoV2, though rarely transmitted to the fetus, can break the BSCF barrier by infecting epithelial cells expressing ACE2 receptor (Pellegrini et al., 2020). Compared to the BBB, the tight junctions in BCSFB are more prone to microbial penetration due to the nature of epithelial cells (Redzic, 2011; Dando et al., 2014). The “leaky barriers” of the brain not only allow the invasion of viruses, but also are amenable for invasion by the peripheral immune components and secretory proteins, which can lead to an altered redox state in the brain. The dysfunctional BBB has been implicated in a number of neurodevelopmental disorders such as seizures, epilepsy, and schizophrenia (Kealy et al., 2020). Viruses such as HSV1 and CMV which are latent upon entry in fetuses, can reactivate postnatally and compromise the permeability of BBB by activating microglia and mounting inflammatory response, resulting in massive influx of other signaling molecules and peripheral immune components into the brain. This initiates a string of pernicious events ultimately leading to decline of brain functions such as cognition and memory. Thus, it would be interesting to compare and contrast different viral neuroinflammatory signatures caused by minor changes in barrier composition vs. total barrier breakdown. Identifying mechanisms as to how the BBB can repair itself will also have implications in designing effective intervention strategies.

## Molecular Basis of Virus-Induced Neurodevelopmental Disorders

**FIGURE 3:**
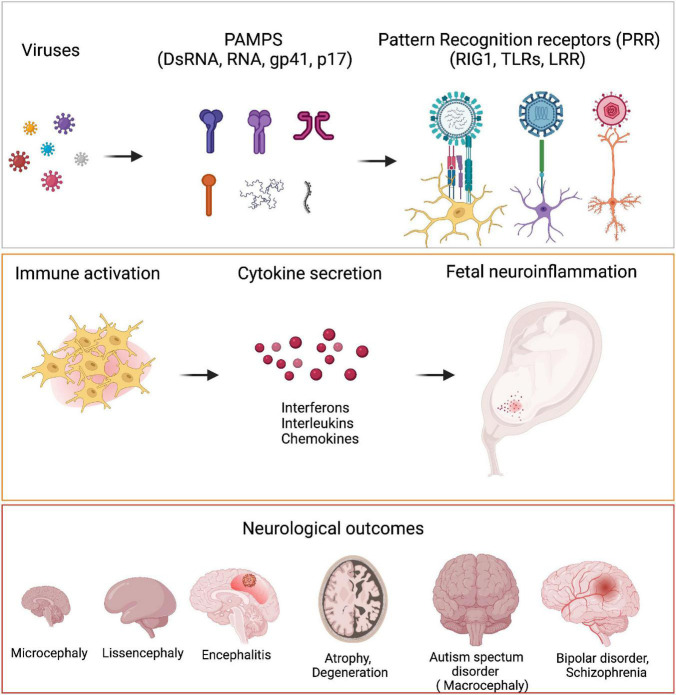
Viruses and neurological outcomes. Viruses have different Pathogen associated molecular pattern (PAMPs) which are sensed by the host cells through their Pattern Recognition Receptors (PRRs). Binding of PAMPs to PRRs leads to host cells secreting different cytokines like IFNα, IL6, IL4, etc., to generate an inflammatory response. PRRs are present on microglia, neurons and astrocytes. During neurodevelopment, these cells secrete several cytokines when they are triggered by viral PAMPs, that bring about detrimental consequences in terms of neurodevelopment. Fetal exposure to pro-inflammatory cytokines in high levels can manifest as neurodevelopmental disorders like microcephaly, lissencephaly, encephalitis, ASD as well as several neuropsychiatric disorders like bipolar disorder and schizophrenia.

How do distinct pathogens manifest different pathological outcomes in terms of neurological disorders? Small motifs called Pathogen associated molecular patterns (PAMPs) unique to each pathogen is sensed by pattern receptors (PRR) such as Toll like receptor (TLRs), which trigger different inflammatory reactions. These in turn translate into different developmental abnormalities (Figure 3).

When there is a productive infection, the maternal immune system is activated, resulting in systemic inflammation, mediated by cytokine release. While the inflammatory cytokines can cross the placental and BBB barriers and are thought to be protective, they can also have detrimental effects (Banks et al., 1995). Proinflammatory cytokine signatures are frequently linked to a variety of neurological disorders including microcephaly, cerebral palsy, schizophrenia and ASD (Ratnayake et al., 2013). Direct injection of pro-inflammatory cytokines such as IL1β and TNFα into the brains of mice, for example, resulted in social withdrawal, altered cognition and diurnal rhythm. In contrast, Insulin Growth factor 1 or IL10 injections attenuated the sickness behavior (Bluthé et al., 2000) alluding to the role of balance in pro- and anti-inflammatory cytokines in orchestration of behavior (Munshi et al., 2019). Such association of cytokines with sickness behavior has also been observed in humans, especially in individuals diagnosed with depression, schizophrenia and autism. Individuals with these neurological conditions exhibit elevated serum anti-inflammatory cytokines such as IL6, IL2R, IFN, IL13, TGF, and IL1 (Knuesel et al., 2014; Xu et al., 2015; Jiang et al., 2018). IL6, the most commonly secreted cytokine during infection, has the potential to alter the cognitive behavior of the progeny to an extent that there is a negative correlation between maternal IL6 and the memory of young children (Smith et al., 2007; Wu et al., 2015; Rudolph et al., 2018). Similarly, elevated levels of CXCL8 (IL8), and TNFα as well as the acute phase protein C-reactive protein (CRP) in maternal serum are linked to an increased risk of schizophrenia in offspring (Buka et al., 2001; Brown et al., 2004). Notably, the interplay between pro-inflammatory cytokines such as TLR4, IL1, IL6 and TNFα released upon maternal infection during pregnancy and preterm births is well established (Pandey et al., 2017). Increased secretion of cytokines such as IL8, IL1β and CRP have been linked with microcephaly, ventriculomegaly and low intelligence quotient (Dammann and O’Shea, 2008; Leviton et al., 2016). Thus, the neurological consequences of many viral infections may be related to the cytokines released rather than the viruses themselves.

In fact, the timing of maternal infection is crucial to the neurodevelopmental outcomes (Solek et al., 2018). Mimicking viral infection by the use of poly I:C at gestation day (GD) 9 or GD17 (early vs. mid stage gestation) in mice showed that while GD 9 infected mice off-springs showed defective spatial exploration, GD17 infected offsprings displayed preservative behavior reflecting autism (Meyer et al., 2006). Similarly, immune challenge in early-mid gestational period leads to increased activation of TNFα and IL10 which is associated with increased postnatal apoptosis (Meyer et al., 2006). Thus immunological challenges at different times of prenatal development may have adverse but variable neurodevelopmental manifestations (Meyer et al., 2007). Therefore, the local immune response in the brain has to be stringently modulated to prevent a huge cytolytic damage, since majority of the cells in CNS (neurons) are non-renewable and terminally differentiated.

## Inflammatory Signatures of Viruses

Do all viral infections elicit similar cytokine profiles? Interestingly, all viruses tend to elicit similar pro-inflammatory cytokines. However, each virus elicits different responses in different cell types based on the type of receptors (Table 1). Upon infection, maternal immune activation produces significant amounts of pro-inflammatory cytokines, many of which including IL6, TNFα, IL10 and IL1β can be detected in the fetal brain (Meyer et al., 2009). This occurs due to the response of different CNS cell types such as neurons, astrocytes and glial cells to the cytokines and infections. Most of these cells have specialized surface receptors such as TLRs (Okun et al., 2011), retinoic acid inducible gene I (RIG) like receptors (Loo and Gale, 2011), mitochondrial antiviral signaling (MAVS) (Nair and Diamond, 2016) and cytokine receptors (Perry et al., 2010). However, differences in the pathogen spread and persistence varies based on the expression levels of each of these immune receptors, expression of innate immune genes and the extent of IFN1 response (Cho et al., 2013). For instance, early cortical neurons which have lower levels of RIG1 are more permissive to WNV and ZKV while in hippocampal neurons high basal expression of type I IFN can restrict varicella virus (Cho et al., 2013; Kennedy et al., 2015). Although HSV1 and ZKV deplete neural progenitor pools and cause a similar phenotype, namely microcephaly, they engage different molecular mechanisms. HSV1 perturbs neuroepithelial polarity and is more severe, while ZKV affects neural progenitor cells without altering the polarity. Furthermore, the sensitivity of ZKV and HSV1 to IFN1 significantly varies, with HSV1 being able to neutralise IFNb unlike ZKV (Krenn et al., 2021).

The predominant host response mechanism that is triggered by several viral infections is the activation of microglia. Fetal microglia differ from adult microglia in their morphology and gene expression profiles (Ginhoux et al., 2013; Kracht et al., 2020). Maternal immune activation with poly I:C in mice revealed that the offspring had an early push toward a more mature microglial developmental state, with a number of autism susceptibility genes differentially expressed (Ozaki et al., 2020). Thus, when maternal immune activation occurs at early gestation, the changes can be sustained in microglia for a longer duration resulting in rewired neural circuits. This rewiring is linked to behavioral defects seen postnatally. While an increased number of activated microglia is essential to stave off infectious agents, a prolonged activation is detrimental leading to neurodevelopmental disorders (Czeh et al., 2011). This is not due to an increase in microglia, but because their immune response is skewed toward a pro-inflammatory state, thereby exposing the fetal and postnatal brain to neuronal loss (Y. S. Kim and Joh, 2006). Once activated, microglia can secrete complement components, the uncontrolled secretion of which could result in abnormal synaptic pruning. This is well exemplified by the fact that the injection of the mouse with poly I:C triggered sustained complement subcomponent C1q secretion in the prefrontal cortex of offspring which often coats the synapse to be eliminated (Han et al., 2017). Notably, mice defective for C1q and CX3CR1 exhibit enhanced excitatory synaptic connectivity similar to those observed in subsets of ASD patients (Chu et al., 2010; Paolicelli et al., 2011; Fagan et al., 2017). The sequestration or inactivation of the complement cascade employed by viruses as an evasion strategy could thus play an indirect role in manifestation of neurodevelopmental disorders (Stoermer and Morrison, 2011).

The source of cytokines in brain is not limited to the resident immune cells but includes immune cells that migrate to the brain guided by chemokine secretion. These peripheral immune cells additionally contribute to an increased production of cytokines in the brain. For example, CXCL10 is overexpressed during a flaviviral infections such ZKV, DENV and WNV and attracts CD-8+ T-cells as a protective mechanism (Klein et al., 2005). However, too much of CXCL10 causes an increase in intracellular calcium levels and triggers apoptosis (Sui et al., 2006). Other chemokines such as CCL4, CCL5 are also significantly upregulated while CCL2 and CXCL8 are significantly downregulated. Interestingly, latent infections can also produce these chemokines which explains the immune infiltration seen in certain cases (Melchjorsen et al., 2003). While the interaction of peripheral immune cell signaling with microglia during viral infections confers protection, it can indirectly damage CNS through synaptic stripping and neuronal death by stimulating neurons to produce CCL2, which acts as a receptor for microglia to phagocytose them (Di Liberto et al., 2018; Moseman et al., 2020).

Transcriptome profiles of host immune cells post-viral infections reveal the upregulation of interferon-stimulated gene family (ISGs) and cell type specific pro-inflammatory genes. For example, CMV-infected pericytes show an upregulation of RANTES, IL6, IL7, IL11, and cyclooxygenase 2 (COX-2) (Cheeran et al., 2001; Zhu et al., 2001). Infected astrocytes predominantly express CCL2 and show TGFβ activation while microglia produce TNFα, IL6 alongside CXCL10, CCL2, CCL3, and CCL5. In such instances, production of anti-inflammatory cytokines such as IL10 is paramount to negate the detrimental consequences of neuroinflammation. This is reflected by the association of genetic variation in IL10 gene with altered HCMV disease occurrence following allogeneic stem cell transplantation and HIV co-infection (Sezgin et al., 2010).

The common presumption that increased production and release of pro-inflammatory cytokines into the fetus can cause brain damage has now been refined. A slender shift in the excess pro- or anti-inflammatory cytokines during an infectious response is sufficient to disrupt normal brain development (Deverman and Patterson, 2009). Contrarily, a uniform change in the expression of pro and anti-inflammatory cytokines such as IL6 and IL10, do not alter post-natal abnormalities, as observed in mice (Meyer et al., 2009). Importantly, viral genomes constantly and rapidly evolve to evade host immune surveillance, resulting in viral proteins mimicking and/or degrading critical immune modulatory signaling pathways. As a case in point, during the viral lytic cycle, CMV produces a functional ortholog of IL10 (UL111A, vIL10) that can suppress a number of innate and adaptive host immune responses including pro-inflammatory cytokine secretion (Jenkins et al., 2004). In the case of ZKV, the RdRP NS_5_ protein binds to and degrades STAT_2_ which is essential for IFN1 response (Kumar et al., 2016). HSV_1_ on the other hand uses the Infected Cell Protein 0 to engage with the host proteasome pathway to degrade Interferon-Stimulated Gene (ISG) products (Van Sant et al., 2001). Additionally, HSV1 prevents the phosphorylation of eukaryotic initiation factor 2, required for translation, by blocking Protein kinase R and recruiting protein phosphatase 1a by the viral protein ICP_34.5_ (Li et al., 2011). DNA viruses, specifically Herpesviruses and Poxviruses, circumvent interferon response by making their own soluble viroceptors/virokines, which can intercept the activities of host cytokines by sequestering them (Smith and Kotwal, 2001). This is exemplified by the binding of the poxvirus protein B8R to IFNγ which attenuates the inflammatory response (Johnston and McFadden, 2003). Emulating this, IFNγ peptide mimetics have been engineered which can circumvent the binding by B8R and be used as an antiviral therapeutic (Ahmed et al., 2005).

## Sexual Dichotomy in Neuroinflammation and Neurological Outcomes

Gender differences in the severity and prevalence of different viral infections are another confounding factor in determining the outcomes of inflammatory responses (Klein and Flanagan, 2016; Mallard et al., 2019). Studies suggest that female fetuses are more resistant to intra-uterine stress and that the male offspring’s immunological homeostasis is particularly affected by maternal immune activation (Goldenberg et al., 2006; Zager et al., 2013). This sexual dimorphism adds complexity to neurological outcomes (Nelson and Lenz, 2017). Males have been shown to be more susceptible to ASD, ADHD, schizophrenia, and bipolar disorder than females (Werling and Geschwind, 2013; Werling, 2016). Males have a faster microglial maturation pathway, which is connected with differential gene expression of immune-related genes (Hanamsagar et al., 2017). While the inherent differences between the male and the female immune systems such as elevated Type I IFN response, T cell numbers, TLR3 response could play a role (Chavez-Valdez et al., 2019), it remains unclear if the transmission risks are the same between the male and the female progeny. Furthermore, differences in immunological regulation between males and females due to glucocorticoid-stimulated cytokine release may also contribute to the sexual dichotomy of the neuroendocrine axis (Bailey et al., 2003; Silverman et al., 2005; Duma et al., 2010). A sustained increase in cytokine production can cause the pituitary to secrete adrenocorticotropic hormone (ACTH), which causes the adrenal gland to release cortisol. Cortisol levels above a certain threshold can harm the hippocampus, affecting learning and memory and promote atrophy in the HPA, hippocampus, and amygdala (Sroykham and Wongsawat, 2019).

## Conclusion and Outlook

Understanding the role of inflammation in neurodevelopmental disorders has now opened up avenues for targeting the immune system of patients with neuropsychiatric disorders such as bipolar disorder, ASD and schizophrenia. For example, the two drugs risperidone and aripiprazole now approved for treatment to improve irritability in ASD and schizophrenia have shown to possess anti-inflammatory effects (Juncal-Ruiz et al., 2018). Likewise use of anti-inflammatory drugs such as minocycline and pioglitazone are being explored to improve irritability and depressive behaviors (Rosenblat, 2019). Thus, a systematic approach to characterizing the molecular and cellular effects of these antipsychotic drugs on inflammation, as well as correlating their clinical response, will pave the way for effective combinatorial therapies, in case of neurodevelopmental disorders with immune dysregulation. Likewise, specific non-steroidal anti-inflammatory drugs (NSAID) such as naproxen have been shown to be effective against ZKV entry (Pan et al., 2018), Influenza virus (Dilly et al., 2018) and reactivation of HSV-1 at least in cellular models. However, this use needs to be exercised with caution since NSAIDs can have diverse effects in addition to their modulation of microglial activation (Ajmone-Cat et al., 2010).

Prenatal exposure to maternal immune activation has been recognized as a risk factor for adverse neurological outcomes by a number of preclinical and epidemiological studies. However, an in depth molecular understanding of how the prenatal exposure to immune activation results in offspring’s neuronal and cognitive impairments in different stages of gestation is lacking. Targeting multiple inflammatory indicators at various times during pregnancy after viral infections will be needed for the development of successful neurodevelopmental disorder therapies. For example, most of the viral infections or neurological diseases show elevated levels of TNFα. Thus intuitively neutralizing this cytokine could ameliorate or prevent the disorder. However, studies thus far have reported discordant findings in this regard. While in murine CMV model and WNV infection model, TNFα antagonist indeed rectified cerebellar abnormalities and developmental gene expression, it has been found to augment Multiple Sclerosis phenotype in humans by increasing peripheral and CNS autoimmunity (Sicotte and Voskuhl, 2001; Shrestha et al., 2008). Although acute infection can be cleared by drug treatment, the extant immune responses, even after pathogen clearance, could cause long-term psychiatric and neurocognitive issues in survivors (Klein et al., 2017). Since many neurodevelopmental disorders have intermediate phenotypes and are subjective, the adoption of multiple complementary methods such as neurochemical investigations and cytokine profiles for accurate diagnosis is necessary. In this regard, use of a powerful platform such as 3D brain organoids with an integrated neuroendocrine and neuro-immune axis should aid to unravel how different viral infections can induce diverse immune gene expression programs in a spatiotemporal manner (Krenn et al., 2021).

Despite the fact that a link between intrauterine viral infections and neurodevelopmental disorders has been established, our ability to prevent or correct such abnormalities using pharmaceutical interventions has been limited. Several studies have shown that regardless of the virus, maternal immune activation and subsequent inflammatory response may be a key determinant of neurological outcomes. Therefore, elucidating the gestation stage specific and sex specific effects of viral infection, as well as inflammatory response codes for various viruses would allow for the development of early diagnosis and intervention strategies for neurodevelopmental disorders.

## Author Contributions

PLC designed the structure and contents of the review. SG and PLC prepared the figures and wrote the manuscript. Both authors contributed to the article and approved the submitted version.

## Conflict of Interest

The authors declare that the research was conducted in the absence of any commercial or financial relationships that could be construed as a potential conflict of interest.

## Publisher’s Note

All claims expressed in this article are solely those of the authors and do not necessarily represent those of their affiliated organizations, or those of the publisher, the editors and the reviewers. Any product that may be evaluated in this article, or claim that may be made by its manufacturer, is not guaranteed or endorsed by the publisher.

## Abbreviations

ASD: autism spectrum disorder
BBB: Blood Brain Barrier
BCSFB: Blood CSF Barrier
CMV: Cytomegalovirus
CNS: central nervous system
CSF: cerebrospinal fluid
HSV: Herpes Simplex Virus
HIV: human immunodeficiency virus
HTLV-1: human T-lymphotropic virus 1
IFN: interferon
IL: interleukin
JEV: Japanese encephalitis virus
WNV: West Nile virus
ZKV: Zika virus.
