# Corrigendum: Intrauterine Viral Infections: Impact of Inflammation on Fetal Neurodevelopment

**DOI:** 10.3389/fnins.2021.817697

**Published:** 2021-12-10

**Authors:** Sourav Ganguli, Pavithra L. Chavali

**Affiliations:** ^1^CSIR-Center for Cellular and Molecular Biology, Hyderabad, India; ^2^Academy of Scientific and Innovative Research (AcCSIR), Ghaziabad, India

**Keywords:** autism spectrum disorder (ASD), blood brain barrier (BBB), blood CSF barrier (BCSFB), microcephaly, neurodevelopment, inflammation, cytokines

In the original article, in the section “Inflammatory signature of viruses”, we have used “measles virus” instead of “varicella virus” in the following sentence: “For instance, early cortical neurons which have lower levels of RIG1 are more permissive to WNV and ZKV while in hippocampal neurons high basal expression of type I IFN can restrict measles virus (Cho et al., 2013).”

Varicella is the correct term referred to in the Kennedy et al. The corrected sentence appears below:

“For instance, early cortical neurons which have lower levels of RIG1 are more permissive to WNV and ZKV while in hippocampal neurons high basal expression of type I IFN can restrict varicella virus (Cho et al., 2013; Kennedy et al., 2015).”

In the original article, the references for Kennedy et al. (2015), Kracht et al. (2020), and Krenn et al. (2021) were incorrectly placed. The corrected first and second paragraph in the section “Inflammatory signature of viruses” is below.

Do all viral infections elicit similar cytokine profiles? Interestingly, all viruses tend to elicit similar pro-inflammatory cytokines. However, each virus elicits different responses in different cell types based on the type of receptors (Table 1). Upon infection, maternal immune activation produces significant amounts of pro-inflammatory cytokines, many of which including IL6, TNFα, IL10 and IL1β can be detected in the fetal brain (Meyer et al., 2009). This occurs due to the response of different CNS cell types such as neurons, astrocytes and glial cells to the cytokines and infections. Most of these cells have specialized surface receptors such as TLRs (Okun et al., 2011), retinoic acid inducible gene I (RIG) like receptors (Loo and Gale, 2011), mitochondrial antiviral signaling (MAVS) (Nair and Diamond, 2016) and cytokine receptors (Perry et al., 2010). However, differences in the pathogen spread and persistence varies based on the expression levels of each of these immune receptors, expression of innate immune genes and the extent of IFN1 response (Cho et al., 2013). For instance, early cortical neurons which have lower levels of RIG1 are more permissive to WNV and ZKV while in hippocampal neurons high basal expression of type I IFN can restrict varicella virus (Cho et al., 2013; Kennedy et al., 2015). Although HSV1 and ZKV deplete neural progenitor pools and cause a similar phenotype, namely microcephaly, they engage different molecular mechanisms. HSV1 perturbs neuroepithelial polarity and is more severe, while ZKV affects neural progenitor cells without altering the polarity. Furthermore, the sensitivity of ZKV and HSV1 to IFN1 significantly varies, with HSV1 being able to neutralise IFNb unlike ZKV (Krenn et al., 2021).

The predominant host response mechanism that is triggered by several viral infections is the activation of microglia. Fetal microglia differ from adult microglia in their morphology and gene expression profiles (Ginhoux et al., 2013; Kracht et al., 2020). Maternal immune activation with poly I:C in mice revealed that the offspring had an early push toward a more mature microglial developmental state, with a number of autism susceptibility genes differentially expressed (Ozaki et al., 2020). Thus, when maternal immune activation occurs at early gestation, the changes can be sustained in microglia for a longer duration resulting in rewired neural circuits. This rewiring is linked to behavioral defects seen postnatally. While an increased number of activated microglia is essential to stave off infectious agents, a prolonged activation is detrimental leading to neurodevelopmental disorders (Czeh et al., 2011). This is not due to an increase in microglia, but because their immune response is skewed toward a pro-inflammatory state, thereby exposing the fetal and postnatal brain to neuronal loss (Y. S. Kim and Joh, 2006). Once activated, microglia can secrete complement components, the uncontrolled secretion of which could result in abnormal synaptic pruning. This is well exemplified by the fact that the injection of the mouse with poly I:C triggered sustained complement subcomponent C1q secretion in the prefrontal cortex of offspring which often coats the synapse to be eliminated (Han et al., 2017). Notably, mice defective for C1q and CX3CR1 exhibit enhanced excitatory synaptic connectivity similar to those observed in subsets of ASD patients (Chu et al., 2010; Paolicelli et al., 2011; Fagan et al., 2017). The sequestration or inactivation of the complement cascade employed by viruses as an evasion strategy could thus play an indirect role in manifestation of neurodevelopmental disorders (Stoermer and Morrison, 2011).

In the original article, the reference for Meyer et al. (2009) was incorrectly written as Kang et al., 2011. A correction has been made to the section “Inflammatory signature of viruses,” paragraph 5. The corrected paragraph is below.

The common presumption that increased production and release of pro-inflammatory cytokines into the fetus can cause brain damage has now been refined. A slender shift in the excess pro- or anti-inflammatory cytokines during an infectious response is sufficient to disrupt normal brain development (Deverman and Patterson, 2009). Contrarily, a uniform change in the expression of pro and anti-inflammatory cytokines such as IL6 and IL10, do not alter post-natal abnormalities, as observed in mice (Meyer et al., 2009). Importantly, viral genomes constantly and rapidly evolve to evade host immune surveillance, resulting in viral proteins mimicking and/or degrading critical immune modulatory signaling pathways. As a case in point, during the viral lytic cycle, CMV produces a functional ortholog of IL10 (UL111A, vIL10) that can suppress a number of innate and adaptive host immune responses including pro-inflammatory cytokine secretion (Jenkins et al., 2004). In the case of ZKV, the RdRP NS_5_ protein binds to and degrades STAT_2_ which is essential for IFN1 response (Kumar et al., 2016). HSV_1_ on the other hand uses the Infected Cell Protein 0 to engage with the host proteasome pathway to degrade Interferon-Stimulated Gene (ISG) products (Van Sant et al., 2001). Additionally, HSV_1_ prevents the phosphorylation of eukaryotic initiation factor 2, required for translation, by blocking Protein kinase R and recruiting protein phosphatase 1a by the viral protein ICP_34.5_ (Li et al., 2011). DNA viruses, specifically Herpesviruses and Poxviruses, circumvent interferon response by making their own soluble viroceptors/virokines, which can intercept the activities of host cytokines by sequestering them (Smith and Kotwal, 2001). This is exemplified by the binding of the poxvirus protein B8R to IFNγ which attenuates the inflammatory response (Johnston and McFadden, 2003). Emulating this, IFNγ peptide mimetics have been engineered which can circumvent the binding by B8R and be used as an antiviral therapeutic (Ahmed et al., 2005).

The authors apologize for this error and state that this does not change the scientific conclusions of the article in any way. The original article has been updated.

## Publisher's Note

All claims expressed in this article are solely those of the authors and do not necessarily represent those of their affiliated organizations, or those of the publisher, the editors and the reviewers. Any product that may be evaluated in this article, or claim that may be made by its manufacturer, is not guaranteed or endorsed by the publisher.
